# Factors Associated With Quality of Life in Patients With Venous Leg Ulcers in a Community Setting: A Cross‐Sectional Study

**DOI:** 10.1002/hsr2.71441

**Published:** 2025-11-10

**Authors:** Jodi Christine McDaniel, Bohyun Kim, Dina Rose McGowan, Alai Tan

**Affiliations:** ^1^ College of Nursing The Ohio State University Columbus Ohio USA; ^2^ College of Nursing Inje University Busan South Korea; ^3^ Clinical Research Center The Ohio State University Wexner Medical Center Columbus Ohio USA

**Keywords:** pain, psychological stress, quality of life, tobacco use, venous leg ulcers

## Abstract

**Background and Aims:**

Quality of life is a well‐established determinant of physical and mental health outcomes in the general population. However, limited research has specifically examined factors influencing quality of life in individuals with venous leg ulcers (VLUs) using disease‐specific measures. This study aimed to identify the key determinants of quality of life in individuals with VLUs and to examine the most salient predictors within this population.

**Methods:**

A cross‐sectional, correlational study was conducted between 2019 and 2024 at a clinical research center affiliated with a major university medical center in the USA. A total of 93 community‐dwelling, middle‐aged and older adults with VLUs participated in the study. Disease‐specific quality of life was measured using the VEINES‐QOL/Sym questionnaire. A multiple linear regression was used to identify the potential predictors of the VEINES QOL/Sym.

**Results:**

Several factors emerged as significant predictors of reduced quality of life. Participants who were middle‐aged, had comorbid atherosclerosis or heart failure, were current smokers, or reported greater ulcer pain, perceived stress, or venous disease severity were more likely to experience diminished quality of life. Among these, ulcer pain, perceived stress, and current tobacco use were strongest predictors, with ulcer pain identified as the most powerful determinant of both impaired quality of life and increased symptom severity.

**Conclusions:**

These findings highlight the complex interaction of physical, psychological, and behavioral factors, underscoring the need for targeted interventions to better manage pain, stress, and tobacco use. Despite the significant psychosocial impact of VLUs, current clinical guidelines do not adequately address these factors, suggesting a need for revisions to improve patient‐centered care. Interventions targeting these modifiable factors are warranted to improve outcomes in this vulnerable population.

## 1 Introduction

Venous leg ulcers (VLUs) are open skin lesions in the lower extremities resulting from chronic venous insufficiency and venous hypertension [1]. As the most prevalent type of leg ulcer, VLUs account for approximately 80% of cases worldwide [2]. In the USA, VLUs affect 4% of individuals over the age of 65, with prevalence expected to rise due to the aging population [3]. Recurrence rates are notably high, with 22% of cases occurring within 3 months post‐healing, over 50% within a year, and nearly 80% within 3 years [4, 5]. The chronic nature and frequent recurrence contribute to a significant economic burden, with annual U.S. healthcare costs estimated at $2.5 billion [6]. Additionally, VLUs result in the loss of an estimated 4.6 million workdays each year [6].

Beyond the financial burden, patients with VLUs often struggle with excessive wound exudate, unpleasant odor, persistent pain, and restricted mobility, all of which can hinder daily activities [1, 7]. Managing VLUs typically requires numerous clinic visits over an extended period, creating transportation and employment challenges [6]. Additionally, odorous drainage, persistent pain and restricted mobility can lead to social isolation, emotional distress, and reduced participation in social activities [8, 9]. The complex interplay of these factors can profoundly impact a patient's quality of life, which the World Health Organization defines as “an individual's perception of their position in life within the context of the culture and value systems in which they live, as well as in relation to their goals, expectations, standards, and concerns” [10].

Extensive evidence links a lower quality of life to negative mental and physical health outcomes [11, 12]. Gaining a deeper understanding of the specific factors influencing quality of life in patients with VLUs could guide the development of more comprehensive care plans that integrate targeted strategies for improvement. Enhancing quality of life may, in turn, support VLU healing and overall patient well‐being [13, 14].

Previous studies on VLU management have primarily focused on wound‐specific treatments aimed at promoting healing [15, 16, 17]. While such research is essential for advancing wound care, there remains a notable gap in understanding the factors that influence quality of life among individuals with VLUs. In particular, few studies use disease‐specific instruments to measure quality of life in this population [13, 18, 19, 20]. Disease‐specific quality of life measures are more effective than generic ones in detecting treatment effects and tracking changes over time in patients with the same condition [21, 22]. The VEnous INsufficiency Epidemiological Study Quality of Life/Symptom (VEINES‐QOL/Sym) questionnaire is a validated, disease‐specific tool designed to assess quality of life in patients with chronic venous disease. Its targeted approach makes it particularly well‐suited for evaluating the impact of VLUs on daily functioning and overall well‐being [23].

To better understand the needs and preferences of patients with VLUs and improve their care, this study aimed to: (1) assess perceived quality of life in community‐dwelling VLU patients using the VEINES‐QOL/Sym questionnaire, (2) identify factors influencing their quality of life, and (3) determine key predictors of quality of life in this population.

## 2 Methods

### 2.1 Research Design and Participants

This cross‐sectional correlational study analyzed quality of life, sociodemographic, perceived stress, and pertinent clinical data collected at baseline from participants enrolled between 2019 and 2024 in a randomized clinical trial (RCT) designed to assess the effects of oral omega‐3 fatty acid supplementation on VLU healing over 4 months. In brief, 93 patients with VLUs were recruited from local ambulatory clinics affiliated with a large academic medical center in the U.S. Inclusion criteria included men and women aged 45 years or older who were able to speak and read English or Spanish and provide written informed consent. Eligible participants also had to have a clinician‐diagnosed VLU that: (1) measured between 2 and 60 cm²; (2) had been present for at least 4 weeks; and (3) was being treated with standard care, including single‐ or multi‐layer compression bandaging. Exclusion criteria included: (1) fish allergy; (2) use of corticosteroids or selective COX‐2 inhibitors; 3) use of nonsteroidal anti‐inflammatory drugs more than twice a week; (4) chemotherapy within 6 months of enrollment; (5) HbA1c > 12%; and (6) VLUs complicated by cellulitis, exposed tendon, or bone. A comprehensive description of the RCT protocol is described elsewhere [24].

### 2.2 Instruments and Variables

#### 2.2.1 Sociodemographic and Clinical Variables

The sociodemographic data evaluated included age, sex, race, marital status, education level, employment status, and annual income. The comorbidity data of interest included hypertension, hypercholesterolemia, diabetes, asthma, chronic obstructive pulmonary disease, emphysema, pulmonary hypertension, atherosclerosis, atrial fibrillation, heart failure, stroke, osteoarthritis, rheumatoid arthritis, depression, and anxiety. Body mass index (BMI) was calculated as weight (kg)/height (m^2^) and categorized into three groups based on the World Health Organization (WHO) criteria: 18.5–24.9 (normal weight), 25.0–29.9 (overweight), and ≥ 30.0 (obesity) [25]. Lifestyle data included smoking status and alcohol consumption, and the VLU‐specific data included ulcer duration and size.

#### 2.2.2 VEINES QOL/Sym Questionnaire

The primary outcome variable, quality of life, was assessed using the VEINES QOL/Sym questionnaire, a tool specifically designed for patients with chronic venous disease and associated conditions such as VLUs. The VEINES‐QOL/Sym achieved a Cronbach's alpha of 0.80 for both the symptom and quality of life subdomains across five culturally and linguistically diverse populations [23, 26]. It also met test‐retest reliability criteria, with an intraclass correlation (ICC) ≥ 0.75 for both quality of life and symptom scores [23]. The process for developing the questionnaire, including content creation, translation methods and psychometric analysis in patients with chronic venous disease, is available in a previous publication [26].

This self‐report questionnaire consists of 26 items that evaluate the impact of chronic venous disease on quality of life and symptoms from the patient's viewpoint. The questionnaire's 26 items are divided into five domains: physical symptoms, social functioning, psychological well‐being, pain, and cosmetic concerns. Patients answer questions using 2‐point to 7‐point scales that assess intensity, frequency, or level of agreement. The questions about symptoms, daily limitations, and psychological impact refer to the previous 4 weeks [27].

Two summary scores can be calculated from the VEINES‐QOL/Sym questionnaire. The VEINES‐QOL score (25 items) evaluates the overall impact of the condition on a patient's quality of life, while the VEINES‐Sym score (10 items) evaluates the severity of symptoms. These symptoms include nine venous‐related issues, such as heavy legs, aching, swelling, night cramps, burning sensation, restless legs, throbbing, itching, and tingling, which are rated on a 5‐point Likert scale based on frequency (from “every day” to “never”). Leg pain is evaluated separately using a 6‐point scale for intensity (ranging from “very severe” to “none”). An additional item (question 2), “At what time of day is your leg problem most intense”, is excluded from the summary scores as it requires a descriptive response.

Higher scores on both the VEINES‐QOL and VEINES‐Sym scales reflect more favorable outcomes. In recent years, the Bland intrinsic scoring method [23] has been recommended over the original *t*‐score method [27] for scoring. Accordingly, we employed the intrinsic scoring approach developed by Bland et al. [23] to calculate the summary scores for VEINES‐QOL and VEINES‐Sym. Initially, each question was assigned a score ranging from 1 to *k*, where *k* represents the number of response categories. We recoded each item score *i* to (*i*−1)(*k*−1). The final score was then obtained by averaging across all items. To derive the composite VEINES‐QOL and VEINES‐Sym scores, the resulting value was multiplied by 100 and rounded up to the nearest whole number [23]. Detailed information regarding the scoring method is provided elsewhere [23].

#### 2.2.3 Venous Clinical Severity Score (rVCSS)

The revised Venous Clinical Severity Score (rVCSS) is an objective grading tool widely used by vascular specialists to assess the severity of venous conditions and to track clinical outcomes and treatment responses over time [23, 28]. The original VCSS tool was revised to update terminology, simplify its use, and eliminate ambiguities, allowing for clearer and more efficient application [29]. The rVCSS includes both patient‐ and physician‐reported outcomes, evaluating 10 venous disease characteristics, including pain, varicose veins, venous edema, skin pigmentation, inflammation, induration, number of ulcers, ulcer duration, ulcer size, and compliance with compression therapy. Each characteristic is rated on a scale from 0 to 3, where 0 indicates none, 1 indicates mild, 2 moderate, and 3 severe. The scores are then summed, resulting in a total score ranging from 0 to 30, which reflects the overall severity of the condition. The rVCSS is a reliable tool for assessing disease severity in patients with lower extremity venous insufficiency, making it valuable for both clinical practice and research studies [28].

#### 2.2.4 Perceived Stress Scale (PSS)

Perceived stress was measured in the current study because prior research has demonstrated its negative association with quality of life in individuals with multiple chronic conditions [30, 31, 32], including slow‐healing wounds [33, 34, 35]. Our study used the 10‐item Perceived Stress Scale (PSS) to measure stress [36]. All participants self‐administered an electronic version of the PSS at baseline. The PSS asks participants to reflect on their stress over the past month, with responses on a 5‐point Likert scale ranging from never (0) to very often (5). The scale is designed to measure how unpredictable, uncontrollable, and overwhelming individuals find their lives [36], with questions such as, “How often have you felt that you were unable to control important things in your life?” and “How often have you felt difficulties were piling up so high that you could not overcome them?” Scores are totaled and fall in a range from 0 to 40, with higher scores indicating higher perceived stress. Cronbach's alpha for the 10‐item scale has consistently been reported to exceed 0.70 across multiple studies involving clinical populations [37, 38].

#### 2.2.5 Wound Measurement

Each VLU was measured using the noncontact Swift Skin and Wound System [39]. This system uses artificial intelligence (AI) to accurately calculate the wound circumference and progress of healing over time. Swift Medical Inc. is a secure mobile application and cloud‐based system that can be used by healthcare providers and researchers to take photos of wounds and monitor wound status. Swift's AI automatically captures length, width and surface area accurately with their fiducial marker, HealX. The computer vision technology also calibrates images for lighting, color, scale, and 3D structure. Additionally, Swift uses data encryption, secure data hosting, and multi‐factor authentication to protect data. The Swift Skin and Wound System has a reported wound measurement accuracy of over 95% and high interrater reliability [39].

#### 2.2.6 Procedure

The study was approved by the local institutional review board of the participating medical center. Eligible patients with VLUs who expressed interest in participating in the study received a comprehensive explanation of the study's purpose and procedures. They were informed of their right to withdraw from the study at any time. At the baseline visit, after obtaining informed written consent, participants completed the study questionnaires. Self‐reported sociodemographic, comorbidity, and select lifestyle data were also collected. Comorbidity data were validated through comprehensive chart reviews. Research personnel measured the size of each VLU in cm^2^ using the Swift Wound Care App. Study visits were conducted at the Clinical Research Center, located on the main campus of the medical center.

All study data were collected and managed using Research Electronic Data Capture (REDCap) tools [40, 41]. REDCap is a secure, web‐based software platform designed to support data capture for research studies.

#### 2.2.7 Statistical Analysis

Descriptive statistics were used to summarize the characteristics of the study participants; means and standard deviations were calculated for continuous variables, while frequencies and percentages were used for categorical variables. Bivariate analyses were performed to explore the unadjusted associations between participants' characteristics and the VEINES QOL/Sym scores using *t*‐test and one‐way analysis of variance (ANOVA) for categorical variables, and Pearson's correlation for continuous variables.

Additionally, multiple linear regression was used to identify the potential predictors of the VEINES QOL/Sym, after adjusting for other covariates in the model. Independent variables that were included in the regression models were selected from those that were significantly associated with VEINES QOL/Sym in the unadjusted analysis. All statistical tests were two‐sided with a significance level of 0.05. Analyses were conducted using SPSS version 29.0.

#### 2.2.8 Power Analysis

Our sample size (*N* = 93) has 84% power to detect a small‐to‐medium correlation *r* = 0.28 with a two‐sided significance level of 0.05. For a multiple linear regression model, our sample size is able to model VEINES SQL/Sym score with up to nine predictors simultaneously based on the rule of thumb N:P ratio of 10:1 and has 80% power to detect an *R*‐Squared of 0.08 for the regression model with a significance level of 0.05.

## 3 Results

### 3.1 Participants' Characteristics

In this study, a total of 93 patients with VLUs were analyzed. The mean QOL score, as measured by the VEINES‐QOL scale, was 41.1 (SD = 23.7), with a median of 36.0 and a range of 2–91. The mean VEINES‐Sym score was 43.6 (SD = 23.5), with a median of 42.0 and a range of 0–93. Responses to VEINES‐QOL/Sym items related to the impact of VLUs on daily activities indicated that 47 participants (51%) reported their daily activities at home were greatly limited, while 25 (27%) said they were somewhat limited. Similarly, 62 participants (67%) reported reducing the time spent on work or other daily tasks due to VLU‐related symptoms, and 67 (72%) said they accomplished less than they would have liked.

Table 1 presents the participants' characteristics. The mean age of the 93 participants was 62.3 years (SD = 9.0; 45–84 years). Of these, 54 (58.1%) were men, 55 (59.1%) were midlife adults (ages 45–64) [42], 53 (57.0%) self‐identified as White, 51 (54.8%) lived alone, 56 (59.6%) had some college or university education, and 73 (78.5%) were unemployed at the time of the study. The possible number of comorbidities in patients with VLUs ranged from 0 to 15, with the majority (70.9%) having two or fewer comorbidities. The most common comorbid conditions were high blood pressure (53.8%), diabetes (37.6%), and hypercholesterolemia (19.4%). Regarding health behaviors, more than half of the participants reported never smoking (52.1%) and no alcohol consumption (74.5%). Among the 93 patients with VLUs, there was a higher proportion of obese individuals (83.9%) compared to those who were overweight (11.8%) and those of normal weight (4.3%).

**Table 1:** Participant characteristics and VEINES‐QoL/Sym scores (*N* = 93).

Variables	Participants characteristics	VEINES‐QoL	VEINES‐Sym
	Mean ± SD or *n* (%)	Mean ± SD	Mean ± SD
**Demographics**			
Age		(*t* = 2.02, *p* = 0.047)*	(*t* = 2.35, *p* = 0.021)*
Middle‐aged adults ( < 65 years)	55 (59.1%)	37.0 ± 24.1	39.0 ± 24.2
Older adults( ≥ 65 years)	38 (40.9%)	46.9 ± 22.0	50.3 ± 21.1
Gender		(*t* = 0.42, *p* = 0.676)	(*t* = 0.13, *p* = 0.894)
Female	39 (41.9%)	42.3 ± 28.5	44.0 ± 26.0
Male	54 (58.1%)	40.2 ± 19.7	43.3 ± 21.9
Race		(*F* = 0.80, *p* = 0.454)	(*F* = 0.49, *p* = 0.614)
White	53 (57.0%)	43.6 ± 23.9	45.6 ± 23.5
Black	34 (36.6%)	37.0 ± 22.6	41.35 ± 22.9
Others	6 (6.4%)	42.2 ± 28.1	38.50 ± 29.8
Marital status		(*t* = −0.61, *p* = 0.545)	(*t* = −0.24, *p* = 0.811)
Living together	42 (45.2%)	42.7 ± 22.1	44.3 ± 21.3
Living alone	51 (54.8%)	39.7 ± 25.0	43.1 ± 25.4
Education level		(*F* = 0.82, *p* = 0.444)	(*F* = 0.83, *p* = 0.441)
Less than college	32 (34.0%)	38.6 ± 24.1	40.6 ± 22.2
Some college/university graduate	56 (59.6%)	41.4 ± 24.2	44.3 ± 25.1
Above college	5 (5.3%)	53.0 ± 9.1	54.6 ± 3.6
Employment status		(*t* = −0.73, *p* = 0.467)	(*t* = −0.90, *p* = 0.372)
Unemployed	73 (78.5%)	40.1 ± 23.4	42.5 ± 23.1
Employed	20 (21.5%)	44.5 ± 25.0	47.8 ± 25.2
Annual income (*N* = 92)		(*F* = 2.82, *p* = 0.065)	(*F* = 1.18, *p* = 0.312)
Low (Below $14,999)	33 (35.1%)	35.3 ± 20.6	40.1 ± 23.6
Middle ($15,000–$39,999)	27 (28.7%)	39.4 ± 25.3	41.9 ± 25.8
High (Above $40,000)	32 (34.0%)	48.8 ± 24.1	48.7 ± 21.6
**Clinical variables**			
Comorbidities		(*F* = 1.73, *p* = 0.182)	(*F* = 0.59, *p* = 0.558)
0–2 comorbidities	66 (70.9%)	43.0 ± 23.7	44.3 ± 23.4
3–5 comorbidities	25 (26.9%)	38.2 ± 23.5	43.2 ± 24.6
≥ 6 comorbidities	2 (2.2%)	14.0 ± 0.0	26.0 ± 8.5
High blood pressure		(*t* = 1.33, *p* = 0.188)	(*t* = 0.95, *p* = 0.344)
Yes	50 (53.8%)	38.1 ± 23.0	41.5 ± 24.6
No	43 (46.2%)	44.6 ± 24.1	46.1 ± 22.3
High cholesterol		(*t* = −0.08, *p* = 0.940)	(*t* = 0.18, *p* = 0.859)
Yes	18 (19.4%)	41.4 ± 29.4	42.7 ± 28.1
No	75 (80.6%)	41.0 ± 22.3	43.8 ± 22.5
Diabetes		(*t* = 0.07, *p* = 0.948)	(*t* = 0.62, *p* = 0.536)
Yes	35 (37.6%)	40.9 ± 22.7	41.7 ± 23.7
No	58 (62.4%)	41.2 ± 24.4	44.8 ± 23.5
Asthma		(*t* = 0.94, *p* = 0.350)	(*t* = 1.66, *p* = 0.101)
Yes	5 (5.4%)	31.4 ± 21.5	26.8 ± 14.8
No	88 (94.6%)	41.6 ± 23.8	44.6 ± 23.6
COPD		(*t* = 1.07, *p* = 0.287)	(*t *= 0.76, *p* = 0.452)
Yes	7 (7.5%)	31.9 ± 19.2	37.1 ± 14.3
No	86 (92.5%)	41.81 ± 23.92	44.14 ± 24.11
Pulmonary hypertension		(*t* = −0.40, *p* = 0.969)	(*t* = −0.83, *p* = 0.410)
Yes	1 (1.1%)	42.0	63.0
No	92 (98.9%)	41.1 ± 23.8	43.4 ± 23.6
Atherosclerosis		(*t* = 2.05, *p* = 0.043)*	(*t* = 1.27, *p* = 0.206)
Yes	4 (4.3%)	17.8 ± 11.0	29.0 ± 13.9
No	89 (95.7%)	42.1 ± 23.6	44.3 ± 23.7
Arterial fibrillation		(*t* = 0.44, *p* = 0.659)	(*t* = −0.07, *p* = 0.943)
Yes	11 (11.8%)	38.1 ± 30.0	44.1 ± 28.0
No	82 (88.2%)	41.46 ± 22.88	43.55 ± 23.06
Heart failure		(*t* = 2.82, *p* = 0.006)*	(*t* = 1.60, *p* = 0.114)
Yes	14 (15.1%)	25.2 ± 18.4	34.4 ± 26.7
No	79 (84.9%)	43.9 ± 23.5	45.2 ± 22.7
Stroke		(*t* = −0.69, *p* = 0.494)	(*t* = −0.56, *p* = 0.577)
Yes	6 (6.5%)	47.5 ± 28.7	48.8 ± 21.7
No	87 (93.5%)	40.6 ± 23.4	43.3 ± 23.7
Osteoarthritis		(*t* = 0.41, *p* = 0.680)	(*t* = −0.51, *p* = 0.614)
Yes	12 (12.9%)	38.4 ± 20.9	46.8 ± 22.8
No	81 (87.1%)	41.5 ± 24.1	43.1 ± 23.7
Rheumatoid arthritis		(*t* = −0.39, *p* = 0.700)	(*t* = −0.39, *p* = 0.700)
Yes	2 (2.2%)	47.5 ± 48.8	50.0 ± 39.6
No	91 (97.8%)	40.9 ± 23.3	43.5 ± 23.4
Depression		(*t* = 1.36, *p* = 0.176)	(*t* = 0.66, *p* = 0.510)
Yes	9 (9.7%)	30.9 ± 19.5	38.7 ± 17.6
No	84 (90.3%)	42.2 ± 23.9	44.1 ± 24.1
Anxiety		(*t* = 1.94, *p* = 0.056)	(*t *= 2.14, *p* = 0.035)*
Yes	2 (2.2%)	9.5 ± 0.7	9.0 ± 8.5
No	91 (97.8%)	41.8 ± 23.4	44.4 ± 23.2
			
Smoking status		(*F* = 5.99, *p* = 0.004)*	(*F* = 8.35, *p* < 0.001)*
		1 > 3 (Scheffe's)*	1,2 > 3 (Scheffe's)*
Never (1)	49 (52.1%)	47.2 ± 25.6	49.3 ± 24.7
Former tobacco user (2)	13 (13.8%)	38.7 ± 19.9	43.9 ± 19.6
Current tobacco user (3)	31 (33.0%)	23.5 ± 13.0	21.5 ± 13.1
Alcohol consumption, drink/week		(*F* = 0.20, *p* = 0.823)	(*F* = 0.51, *p* = 0.603)
0/week	70 (74.5%)	40.5 ± 24.0	42.8 ± 24.4
1–7 cups/week	18 (19.1%)	41.7 ± 24.1	44.0 ± 22.9
≥ 8 cups/week	5 (5.3%)	47.2 ± 20.9	53.8 ± 8.7
BMI		(*F* = 1.41, *p* = 0.249)	(*F* = 0.61, *p* = 0.547)
Normal	4 (4.3%)	44.5 ± 27.1	44.5 ± 26.8
Overweight	11 (11.8%)	51.9 ± 19.1	50.9 ± 23.8
Obese	78 (83.9%)	39.4 ± 23.9	42.5 ± 23.5
PSS	16.2 ± 7.8		
**Wound specific variables**			
Duration of ulcer (weeks)	81.8 ± 453.0		
Ulcer dimensions (cm^2^)	8.3 ± 10.0		
VCSS	15.5 ± 3.8		
Ulcer pain (*N* = 92)		(*F* = 12.37, *p* < 0.001)*	(*F* = 11.98, *p* < 0.001)*
		1 > 2,3,4 (Scheffe's)*	1 > 3,4 (Scheffe's)*
None (1)	23 (25.0%)	61.7 ± 19.0	61.6 ± 21.0
Mild (2)	25 (27.2%)	39.6 ± 20.7	45.4 ± 19.1
Moderate (3)	26 (28.3%)	35.7 ± 20.8	39.5 ± 22.0
Severe (4)	18 (19.6%)	25.0 ± 20.4	24.1 ± 18.3

Abbreviations: BMI = body mass index, COPD = chronic obstructive pulmonary disease, PSS = perceived stress scale, QoL = quality of life, SD = standard deviation, Sym = symptoms, VCSS = venous clinical severity score, VEINES = VEnous INsufficiency Epidemiological and Economic Study.

* *p* < 0.05.

The mean PSS score was 16.2 (SD = 7.8), indicating moderate stress levels. Additionally, the mean duration of VLUs was 81.8 weeks (SD = 453.0), and the mean ulcer size was 8.3 cm^2^ (SD = 10.0). The VCSS of the participants ranged from 5 to 24, with a mean total score of 15.5 (SD = 3.8), indicating moderate wound severity. Moderate pain was reported by 28.3% of the cases, while mild pain and severe pain were noted among 27.2% and 19.6% of the cases, respectively.

### 3.2 Unadjusted Analysis on Patient Characteristics Associated With VEINES QOL/Sym Scores

Table 1 also provides the differences in VEINES‐QOL/Sym scores according to the study variables. We initially examined the VEINES‐QOL scores. Middle‐aged adults reported lower VEINES‐QOL scores compared to older adults (mean ± SD: 37.0 ± 24.1 vs. 46.9 vs. 22.0, *p* = 0.047), indicating a significant difference in VEINES‐QOL based on age. Patients with atherosclerosis had lower VEINES‐QOL scores compared to those without atherosclerosis (42.1 ± 23.6 vs. 17.8 vs. 11.0, *p* = 0.043). Additionally, patients with heart failure demonstrated lower scores than those without heart failure (25.2 ± 18.4 vs. 43.9 ± 23.5, *p* = 0.006). This suggests that the presence of comorbid conditions such as atherosclerosis and heart failure is associated with poorer quality of life.

The VEINES‐QOL scores varied significantly across smoking status (*F* = 5.99, *p* = 0.004). Scheffe's post hoc test indicated that patients who were current tobacco users had lower VEINES‐QOL scores compared to those who had never smoked (23.5 ± 13.0 vs. 47.2 ± 25.6). Moreover, a statistically significant association with ulcer pain severity was observed (*F* = 12.37, *p* < 0.001). Scheffe's post hoc test revealed that patients without ulcer pain had at least 20 points higher average VEINES‐QOL scores compared to those with mild, moderate, or severe ulcer pain.

Similarly, we investigated the VEINES‐Sym scores by participant characteristics. Consistent with findings for the VEINES‐QOL score, middle‐aged adults had lower VEINES‐Sym scores than older adults (39.0 ± 24.2 vs. 50.3 ± 21.1, *p* = 0.021). Furthermore, patients with anxiety reported lower scores than those without anxiety (9.0 ± 8.5 vs. 44.4 ± 23.2, *p* = 0.035). The scores varied significantly across smoking status (*F* = 8.35, *p* < 0.001), with Scheffe's post hoc test revealing that current tobacco user had lower VEINES‐Sym scores (21.5 ± 13.1) compared to former tobacco users (43.9 ± 19.6) or never smokers (47.2 ± 25.6). A statistically significant association with ulcer pain was noted (*F* = 11.98, *p* < 0.001). Patients without ulcer pain had higher scores than those with moderate or severe ulcer pain, as determined by Scheffe's post hoc test.

Table 2 presents the correlation of participants' characteristics of PSS, duration of ulcer, ulcer dimensions and VCSS with the VEINES‐QOL/Sym scores. A medium‐to‐large negative correlation was identified between PSS scores and both VEINES‐QOL scores (*r* = −0.515, *p* < 0.001) and VEINES‐Sym scores (*r* = −0.447, *p* < 0.001), indicating that higher stress levels are associated with poorer quality of life and more severe symptoms. Additionally, a medium negative correlation was found between the VCSS scores and both VEINES‐QOL scores (*r* = −0.318, *p* = 0.002) and VEINES‐Sym scores (*r* = −0.301, *p* = 0.004), confirming that greater venous clinical severity is associated with lower quality of life and worse symptom severity. However, no significant correlations were observed between the ulcer duration or dimensions and VEINES‐QOL/Sym scores.

**Table 2:** Correlations between participant characteristics and VEINES‐QoL/Sym scores (*N* = 93).

Variables	VEINES‐QoL	VEINES‐Sym
PSS	−0.515* (*p* < 0.001)	−0.447* (*p* < 0.001)
Duration of ulcer, weeks	0.123 (*p* = 0.239)	0.008 (*p* = 0.941)
Ulcer dimensions (cm^2^)	−0.121 (*p* = 0.248)	−0.191 (*p* = 0.067)
VCSS	−0.318* (*p* = 0.002)	−0.301* (*p* = 0.004)

Abbreviations: PSS = perceived stress scale, QoL = quality of life, Sym = symptoms, VCSS = venous clinical severity score, VEINES = VEnous INsufficiency Epidemiological and Economic Study.

* *p* < 0.05.

### 3.3 Multiple Linear Regression Models on Potential Predictors of VEINES‐QOL/Sym Scores

Table 3 displays the findings from the multiple linear regression analysis. Sociodemographic, clinical, and wound‐specific variables were included in the regression model if they exhibited significant differences among participants or showed significant correlations with the VEINES‐QOL/Sym scores. Independent variables for the multiple regression models were selected from variables that were significantly associated with VEINES‐QOL/Sym scores in bivariate analyses, including age, the presence of comorbidities (i.e., atherosclerosis, heart failure, and anxiety), smoking status, ulcer pain, PSS, and VCSS. The Durbin–Watson test statistics, calculated at 1.80 for VEINES‐QOL and 1.71 for VEINES‐Sym, confirmed the independence of residuals. The tolerance values for all variables ranged from 0.38 to 0.84, indicating that none fell below the threshold of 0.10. Additionally, the variance inflation factors (VIFs) for the variable ranged from 1.19 to 2.62, indicating the absence of multicollinearity.

**Table 3:** Multiple linear regression models on factors associated with VEINES‐QoL/Sym scores (*N* = 92).

Variables	VEINES‐QoL				VEINES‐Sym			
	*B*	SE	*β*	*p*	*B*	SE	*β*	*p*
Age								
Middle‐aged adults*	2.1	4.3	0.0	0.626	4.1	4.4	0.1	0.357
Older adults								
Atherosclerosis	−15.5	10.6	−0.1	0.147	−8.8	10.9	−0.1	0.423
Heart failure	−9.0	5.7	−0.1	0.117	0.6	5.8	0.0	0.921
Anxiety	−10.2	14.5	−0.1	0.483	−17.0	14.9	−0.1	0.257
Smoking status								
Never*	−4.9	4.4	−0.1	0.274	−2.9	4.5	−0.1	0.522
Former tobacco user	−**13.0**	**6.1**	−**0.2**	**0.036**	−**18.8**	**6.3**	−**0.3**	**0.004**
Current tobacco user								
Ulcer pain								
None*	−**12.4**	**5.9**	−**0.2**	**0.040**	−9.6	6.1	−0.2	0.120
Mild								
Moderate	−**14.9**	**6.4**	−**0.3**	**0.023**	−**15.2**	**6.6**	−**0.3**	**0.024**
Severe	−**22.1**	**7.6**	−**0.4**	**0.005**	−**28.8**	**7.8**	−**0.5**	< **0.001**
PSS	−**1.0**	**0.3**	−**0.3**	**< 0.001**	−**0.7**	**0.3**	−**0.2**	**0.009**
VCSS	−0.1	0.7	−0.0	0.921	0.4	0.7	0.1	0.579
*F*	7.25 (*p* < 0.001)				6.42 (*p* < 0.001)			
*R* ^2^	0.50				0.47			

Abbreviations: β = standardized coefficients, B = unstandardized coefficients, PSS = perceived stress scale, QoL = quality of life, SE = standard error, Sym = symptoms, VCSS = Venous Clinical Severity Score, VEINES = VEnous INsufficiency Epidemiological and Economic Study

* Reference group. Bold values highlight statistical significance.

The multiple linear regression model for VEINES‐QOL was statistically significant (*F* = 7.25, *p* < 0.001), explaining 50% of the variance. After adjusting for other covariates, current tobacco use, greater ulcer pain, and higher levels of stress were all associated with a poorer quality of life. Similarly, the regression model for VEINES‐Sym was also statistically significant (*F* = 6.42, *p* < 0.001), accounting for 47% of the variance. As with the VEINES‐QOL regression model, current tobacco use, increased pain, and higher stress levels were linked to more severe symptoms after adjusting for other covariates. Severe ulcer pain was the strongest predictor of diminished quality of life and heightened symptom severity in patients with VLUs. Compared to no ulcer pain, severe ulcer pain was associated with a 22.1‐point lower (SE = 7.6, *p* = 0.005) VEINES‐QOL score and a 28.8‐point lower (SE = 7.8, *p* < 0.001) VEINES‐Sym score after adjusting for other covariates in the model. Moderate ulcer pain was linked to a 14.9‐point lower (SE = 6.4, *p* = 0.023) VEINES‐QOL score and a 15.2‐point lower (SE = 6.6, *p* = 0.024) VEINES‐Sym score, and mild ulcer pain was associated with a 12.4‐point lower (SE = 5.9, SD = 0.040) VEINES‐QOL score. Additionally, compared to never smokers, current tobacco use was associated with a 13.0‐point lower (SE = 6.1, *p* = 0.036) VEINES‐QOL score and an 18.8‐point lower (SE = 6.3, *p* = 0.004) VEINES‐Sym score.

## 4 Discussion

Although poorer physical and mental health is strongly associated with reduced quality of life in the general population [43, 44], few studies have specifically examined the primary factors impacting quality of life in individuals with VLUs using disease‐specific measures. Identifying these factors is essential for building evidence that could inform updates to VLU management guidelines, which currently focus primarily on locally applied treatment regimens. Broadening these guidelines to address both physical and psychosocial factors could lead to more holistic and effective management approaches, ultimately improving patients' quality of life and healing outcomes. In this study of community‐dwelling people living with VLUs, greater perceived stress and wound severity were linked to lower quality of life as measured by the VEINES‐QOL/Sym questionnaire. The data further revealed that current tobacco use, increased ulcer pain, and elevated stress levels predicted diminished quality of life, with severe ulcer pain emerging as the strongest predictor of reduced quality of life and heightened symptom severity.

### 4.1 Demographics and Clinical Variables

Although women and older adults are commonly identified as high‐risk groups for VLUs [2, 45, 46], the majority of our participants were men and under the age of 65. However, consistent with existing literature, a large proportion (83.9%) were classified as obese (BMI > 25 kg/m²) [2]. Over half of the participants lived alone, suggesting that they may be solely responsible for their own wound care at home, a pattern that aligns with previous research indicating that many VLU patients rely on themselves or informal caregivers, such as family and friends, for wound management [47, 48].

While participants received direct wound care during clinic visits, they were responsible for managing their care between appointments. This is particularly challenging for older adults, as effective wound care requires physical flexibility, dexterity, and strength—capabilities that commonly decline with age. Additionally, more than three‐quarters of participants were unemployed, and more than one‐third reported an annual income below $15,000. These findings underscore the significant socioeconomic challenges faced by many VLU patients, including financial barriers that may limit access to healthcare resources and support. This aligns with prior research linking lower socioeconomic status to a higher prevalence of VLUs [49, 50].

Nearly all participants were managing up to five chronic conditions in addition to their VLUs, with the most common being hypertension, diabetes, and hypercholesterolemia. This finding is similar to previous research showing that people with VLUs often manage multiple comorbidities, as VLUs frequently develop within a broader context of circulatory and systemic health issues [51]. This emphasizes the importance of a holistic, multidisciplinary approach to care.

Over one‐third of the participants reported current tobacco use, and nearly 14% identified as former users. These findings align with studies showing that tobacco use is common among individuals with chronic wounds, including VLUs [52, 53]. This is concerning, as tobacco use—especially smoking—impairs circulation, reduces tissue oxygenation, and weakens immune function, all of which hinder the healing process [54, 55, 56].

Although few participants reported treatment for depression or anxiety, the mean PSS score of 16.5 indicates moderate stress [36]. This suggests that participants were likely experiencing some pressure or demands, but not at a high‐stress level.

The study's findings highlight the chronic nature of VLUs, with an average duration of 81.8 weeks and a mean wound size of 8.33 cm², indicating larger and more complex ulcers. The average rVCSS score suggests mild to moderate venous disease, characterized by symptoms such as pain, edema, and skin changes. Notably, our sample exhibited higher rVCSS scores than those in similar studies [57, 58, 59], suggesting greater baseline disease severity and underscoring the complexity of VLUs in this population.

### 4.2 Demographic and Clinical Differences in the VEINES‐QOL/Sym Subdomains

Middle‐aged adult patients with VLUs ( < 65 years) reported a lower quality of life compared to older adult patients ( ≥ 65 years) in our study. This may be attributed to the increased demands associated with work, family responsibilities, and financial obligations in midlife. Additionally, middle‐aged adults exhibited a higher symptom burden, as reflected in their symptom domain scores, compared to older adult patients. This suggests that life‐stage factors may significantly influence both quality of life and symptom experiences in patients with VLUs.

In examining clinical variables, we found that patients with atherosclerosis and heart failure reported a lower quality of life compared to those without these conditions. Additionally, patients with anxiety had a higher symptom burden. Differences in quality of life and symptom burden were also evident across tobacco use groups: current smokers reported a significantly lower quality of life compared to never‐smokers and higher symptom burden compared to both past smokers and never‐smokers. These findings align with research showing that smoking and nicotine dependence are associated with poorer quality of life in older adults [60]. Smoking also correlates with increased symptom burden due to its strong association with lower limb venous insufficiency, a major contributor to the development of VLUs [61]. Unfortunately, smoking and other forms of tobacco use are highly prevalent among individuals with VLUs [62].

Among wound‐specific variables, patients with greater ulcer pain reported significantly lower quality of life and a higher symptom burden than those with less intense pain. In studies exploring chronic wounds, including VLUs, pain consistently emerges as a significant factor impacting quality of life. Pain from VLUs often contributes to reduced mobility, sleep disturbances, and emotional distress, all of which lower overall quality of life. Research by Folguera‐Alvarez et al. [63] and Hopman et al. [64] highlights that pain intensifies both physical and psychological limitations, creating a cycle that can hinder wound healing and heighten the risk of conditions like depression and anxiety.

### 4.3 Correlations With Patient Characteristics' and VEINES‐QOL/Sym Scores

Higher perceived stress among study participants was associated with both lower quality of life and increased symptom burden. These findings suggest that as stress levels rise, individuals experience both a significant decline in overall well‐being and an intensification of symptoms. Studies consistently show that individuals with VLUs report higher anxiety levels than healthy controls, likely stemming from factors like persistent pain, social isolation, and concerns over body image [9]. Da Silva et al. [65] found that male patients, in particular, reported social difficulties impacting productivity and personal relationships, which heightened their anxiety as they struggled to maintain daily roles and responsibilities. Similar findings were reported by Moffatt et al., [66] where anxiety in VLU patients was worsened by prolonged treatment, the ulcer's cosmetic impact, and the stigma surrounding the condition.

Similarly, greater venous clinical severity was linked to a decline in quality of life and an increase in symptom distress. These results are consistent with existing research that highlights a significant association between ulcer characteristics, pain intensity, emotional distress, and overall quality of life [7, 9, 58, 63].

### 4.4 Predictors of Quality of Life and Symptom Severity in Patient with VLUs

Our multiple regression analysis identified all levels of ulcer pain, higher levels of stress, and current tobacco use as significant predictors of quality of life in our study participants. Similarly, moderate ulcer pain, severe ulcer pain, higher levels of stress, and current tobacco use were significant predictors of symptom severity. These findings highlight the importance of addressing the factors of pain, stress and tobacco use to improve patient well‐being.

As expected, and in line with our findings, pain has been identified as a significant predictor of quality of life in previous studies of individuals with VLUs. Research consistently shows that higher levels of pain correlate with poorer quality of life in these patients [14, 58, 63, 67]. Chronic wound‐related pain has a profound impact on daily functioning, social interactions, and psychological well‐being, thus worsening the overall health outcomes in this population [68]. These findings highlight the critical need to assess and manage pain to enhance the quality of life for individuals with VLUs. A comprehensive review of validated and reliable pain assessment tools for this population has been previously published [68].

While the study sample had a mean PSS score indicating moderate stress, perceived stress emerged as a key predictor of quality of life. Although research on perceived stress in individuals with VLUs is limited, Walburn et al. [11] reported comparable PSS scores in VLU patients and found that higher stress levels predicted slower wound healing.

Perceived stress is an individual's subjective assessment of stress levels and coping ability [69]. Higher stress is linked to accelerated aging, elevated cortisol, weakened immunity, and increased inflammation, heightening infection risk and slowing wound healing [11, 70, 71, 72]. It also correlates with poor health habits like inadequate sleep, skipped meals, and increased alcohol use [73]. Stress also influences pain perception, with a study of 32 chronic wound patients showing its impact on activity limitations, sleep disturbances, and reduced life enjoyment [71]. Prolonged exposure to elevated stress levels significantly deteriorates quality of life, affecting both physical and mental well‐being over time.

The finding that current tobacco use also predicts reduced quality of life, is likely due to its combined adverse effects on wound healing, pain, and overall health. Tobacco chemicals impair oxygen delivery to tissues and blood circulation, slowing healing and worsening VLU symptoms [54, 61, 74, 75]. Tobacco use also intensifies inflammation and stress, further exacerbating symptoms burden and limiting daily activities [60, 62]. These findings emphasize the need for smoking cessation support as a crucial component of VLU care to improve patient outcomes.

## 5 Strength, Limitations, and Future Research Recommendations

A strength of this study is its comprehensive evaluation of sociodemographic, health, and lifestyle factors and its use of the VEINES‐QOL/Sym tool for accurate quality‐of‐life measurement. Limitations include a relatively small sample size, self‐reported data, which may lead to reporting biases, and the exclusion of factors like frailty, daily activity limitations, loneliness, and cognitive decline, which have been associated with poorer health‐related quality of life in the general older adult population [76]. The impact of blood components on quality of life was also not examined. The cross‐sectional design further limited assessment of treatment effects, care duration, and VLU healing on quality of life predictors, especially pain.

Longitudinal research is needed to examine bidirectional relationships between various factors and quality of life in VLU patients over time. Further investigation into biological factors and mechanisms could clarify their role in psychosocial and physical well‐being. Moreover, while this study suggests that interventions focused on pain management, stress reduction, and tobacco cessation may enhance both quality of life and VLU healing, future research should evaluate the effectiveness of these approaches, both individually and in combination.

## 6 Clinical Implications

We acknowledge that the clinical consequences of reduced quality of life are well established. Nonetheless, our study adds value by drawing attention to the expanding evidence connecting pain, stress, and tobacco use with diminished quality of life in individuals with VLUs. Although these relationships are not entirely new, they remain underrepresented in current clinical guidelines, and consequently, these factors are not consistently assessed by clinicians using validated measures. Assessing pain characteristics associated with VLUs—such as its location, intensity, and effects on physical, emotional, and social well‐being—can significantly enhance treatment strategies tailored to individual patient needs [68]. Similarly, incorporating VLU‐specific stress assessments may provide valuable insights for developing comprehensive, personalized care plans. Tobacco use should also be routinely evaluated, with discussions on evidence‐based cessation strategies for current users. In addition to pharmacological options for pain, stress, and tobacco cessation, clinicians should consider evidence‐based nonpharmacological interventions, such as mindfulness therapy, cognitive behavioral therapy, or music therapy [77, 78, 79]. Despite the significant psychosocial impact of VLUs, current clinical guidelines do not adequately address these factors, highlighting the need for revisions to improve patient‐centered care and overall quality of life.

## 7 Conclusions

This study assessed quality of life in 93 middle‐aged and older adults with active VLUs and identified key predictors. Poor quality of life was more common among middle‐aged adults, those with atherosclerosis or heart failure, current smokers, and individuals with higher VLU pain, stress, or venous severity. Significant predictors included ulcer pain, perceived stress, and tobacco use. These findings highlight the complex interplay of physical, psychological, and behavioral factors, emphasizing the need for targeted interventions—pain management, stress reduction, and tobacco cessation—to improve well‐being and wound healing.

## Author Contributions

**Jodi Christine McDaniel:** conceptualization, investigation, funding acquisition, writing – original draft, supervision, methodology, validation, writing – review and editing, visualization, resources, data curation, project administration. **Bohyun Kim:** conceptualization, methodology, formal analysis, validation, visualization, data curation, writing – review and editing. **Dina Rose McGowan:** project administration, writing – review and editing, conceptualization. **Alai Tan:** conceptualization, writing – review and editing, formal analysis, data curation, validation.

## Ethics Statement

This study was approved by the local institutional review board of the participating medical center (The Ohio State University [OSU] Wexner Medical Center—July 20, 2018; #2018H0261). All study procedures were conducted in accordance with the Declaration of Helsinki. World Medical Association. World Medical Association Declaration of Helsinki: “Ethical Principles for Medical Research Involving Human Participants,” *JAMA* 333, no. 1 (2025):71–74. doi: 10.1001/jama.2024.21972.

## Consent

Written informed consent was obtained from all participants prior to data collection.

## Conflicts of Interest

The authors declare no conflicts of interest.

## Transparency Statement

The lead author, Jodi Christine McDaniel, affirms that this manuscript is an honest, accurate, and transparent account of the study being reported; that no important aspects of the study have been omitted; and that any discrepancies from the study as planned (and, if relevant, registered) have been explained.

## Data Availability

The authors confirm that the data supporting the findings of this study are available within the article or its supplementary materials.
